# Oilcloth sessions as an implementation strategy: a qualitative study in Denmark

**DOI:** 10.1186/s12909-022-03635-w

**Published:** 2022-07-23

**Authors:** Jeanette Wassar Kirk, Nina Þórný Stefánsdóttir, Byron J. Powell, Mette Bendtz Lindstroem, Ove Andersen, Tine Tjørnhøj-Thomsen, Per Nilsen

**Affiliations:** 1 Department of Clinical Research, Copenhagen University Hospital Hvidovre, 2650 Hvidovre, Denmark; 2 Department of Public Health, Nursing, Aarhus University, 8000 Aarhus C, Denmark; 3 Center for Mental Health Services Research, Brown School and School of Medicine, Washington University in St. Louis, St. Louis, MO USA; 4 Emergency Department, Copenhagen University Hospital Hvidovre, 2650 Hvidovre, Denmark; 5 Department of Clinical Medicine, University of Copenhagen, 2200 Copenhagen, Denmark; 6 Department of Health and Social Context, National Institute of Public Health, University of Southern Denmark, 1455 Copenhagen K, Denmark; 7 Department of Health, Medicine and Caring Sciences, Linköping University, 581 83 Linköping, Sweden

**Keywords:** Implementation research, Implementation strategy, Oilcloth session, Alignment, Emergency department

## Abstract

**Background:**

The aim of this study was to explore healthcare professionals, managers, and other key employees’ experiences of oilcloth sessions as a strategy when implementing new emergency departments in Denmark, based on their participations in these sessions. The study addresses the importance of securing alignment in implementation strategies. Too often, this does not get enough attention in the literature and in practice. In this study, alignment among components was achieved in an educational implementation strategy called oilcloth sessions.

**Methods:**

The study is based on participants’ observations of 13 oilcloth sessions and follow-up via 53 semi-structured interviews with the board of directors, managers, and key employees from the present emergency department and different specialty departments. Data were analysed deductively using Biggs and Tang’s model of didactic alignment.

**Results:**

The analysis showed the complexity of challenges when using oilcloth sessions as a strategy when implementing a new emergency department described in terms of three phases and nine main themes (a–i): the preparation phase: (a) preparing individually and collectively, (b) objectives, (c) involving participants, (d) selecting cases; the execution phase: (e) using materials, (f) facilitating the sessions, (g) temporal structures; evaluation: (h) following up on the sessions, (i) adapting to the context.

**Conclusions:**

This study shows that it is important to ensure alignment among elements in implementation strategies. Thus, oilcloth sessions with high alignment are useful if the challenges experienced are to be overcome and the strategy will be experienced as a useful way to support the implementation of a new emergency department from the participants’ point of view. Bigg and Tang's didactic model is useful as an analytical framework to ensure alignment in implementation strategies in general.

## Background

Emergency services in Denmark have changed over the past decade as a result of policy reforms by the Danish Health Authority [1]. These reforms include the establishment and implementation of new emergency departments (ED) (in Danish Fælles Akutmodtagelser or simply FAM). In Denmark, EDs are the primary entry point for about 1 million of the 1.3 million hospitalizations [2] and thereby a linchpin of the acute healthcare system. Centralization of emergency services in Denmark has been advocated politically because it is expected to improve access to specialized facilities and equipment, and reduce the risk of being admitted to the wrong “silo” of highly specialized physicians, which increases the risk of erroneous or missed diagnoses in the first hours of acute hospitalization [3]. Historically, emergency care was often provided according to specialty at different departments in different locations in the hospitals [1, 4, 5]. Specialty emergency medicine was first established as a new specialty in 2017 in Denmark. The experience with physicians specialized in this area is therefore scarce.

The new EDs in Denmark represent an organizational innovation because considerable changes both in the organization’s practices and routines and in the workplace organization and professional relations are required. Innovation involving the implementation of new methods in an organization’s business practices, workplace and/or external relations [6] is important for an organization’s short-term competitiveness and long-term survival [7]. This type of innovation typically relies more on informal processes and relationships between organizational members than on formal processes [8, 9].

The challenges of organizational innovation in healthcare are substantial [10] due to the diversity of the professional disciplines and medical specialties involved [11]. Studies report the importance of adapting the implementation of organizational innovations in healthcare to the local context and the use of appropriate implementation strategies to facilitate their integration [12]. Numerous implementation strategies, defined as “methods or techniques used to enhance the adoption, implementation, and sustainability of a clinical program or practice” have been described and evaluated [13]. Powell and colleagues identify no less than 68 discrete strategies [14]. Discrete implementation strategies are defined as a single approach or technique, such as distributing educational materials to healthcare professionals or informing local opinion leaders, whereas multifaceted implementation strategies combine two or more discrete strategies [15]. Scholars have favoured multifaceted strategies in changing behaviour at an individual, team, organizational, and system levels [16] despite systematic reviews questions the validity of such claims [17, 18].

The current study concerns a new organizational model of an ED that is being constructed in a university hospital in the Capital Region in Denmark. The new ED is expected to be built and operational by spring 2023. It involves setting up a single point of hospital entry for all emergency care patients [3], increasing the number of patient beds from 29 to 92 for hospitalization up to 48 hours, and ensuring continuous presence of specialist physicians [19]. The new ED involves a merger between two existing EDs in the hospital, one affiliated with the department of gastroenterology, and another handling all other acute admittances, including medical diseases and trauma.

The board of directors of the hospital in question has decided on several implementation strategies to facilitate the implementation of the new ED. One strategy involves so-called “oilcloth sessions” (in Danish “voksdug”), a strategy that has been used in a few studies of organizational innovations in Denmark in relation to the reorganization of hospitals [20] and physical environments [21]. Oilcloth sessions is an Danish invention but can be compared with table-tops models internationally, which is a common method in gaming and computer animation [22]. However, oilcloth sessions use of a minor degree of 3D animation. Oilcloth sessions combine elements of three types of implementation strategies: educational (training), restructuring (altering professional roles, physical structures, equipment, etc.) and facilitation (supporting processes) [14, 23, 24].

Oilcloth sessions are a type of learning laboratory [25]. Such laboratories can take different forms, such as simulation-based training focusing on both technical and non-technical skills often used in clinical practice in healthcare [26], participatory ergonomics simulation, which is a method used to design new hospital work systems [27], or virtual labs where technology is used in the form of computer-based simulations [28]. Within social sciences, learning laboratories are understood as “an organized practice in relation to which knowledge is generated and experiments concerning competencies, positions, participation, engagements and reflections are carried out” [25].

Oilcloth sessions as an implementation strategy to facilitate the implementation of an organizational change such as a new ED have not been studied. Further, organizational and structural changes in EDs are a global issue [29]. Thus, knowledge about the challenges or effectiveness of this strategy for implementation purposes or its potential for wider use in healthcare when implementing organizational changes is warranted. Addressing these knowledge gaps, the aim of this study was to explore healthcare professionals, managers, and other key employees’ experiences of oilcloth sessions as a strategy when implementing a new ED in Denmark, based on their participations in these sessions.

## Methods

### Study design

This study is based on a qualitative design, which is particularly suitable to explore deeper understandings of attitudes, experiences, motives, behaviours, and any discrepancies between these [30]. Data included field notes from an ethnographic study and follow-up semi-structured interviews with managers and key employees [31].

### Setting

The study was conducted in Denmark, where the public healthcare system is funded by taxpayers and provides free treatment for primary medical care, hospitals, and homecare services for all citizens. The hospital in the study has around 700 beds, 5000 employees, around 100,000 admissions a year and a catchment area of 517,000 citizens. The board of directors constitutes the top management of the hospital and is supported by 18 clinical departments with separate management teams in charge of clinical, financial, and organizational decisions within the department.

### The implementation strategy: oilcloth sessions

**Fig. 1:**
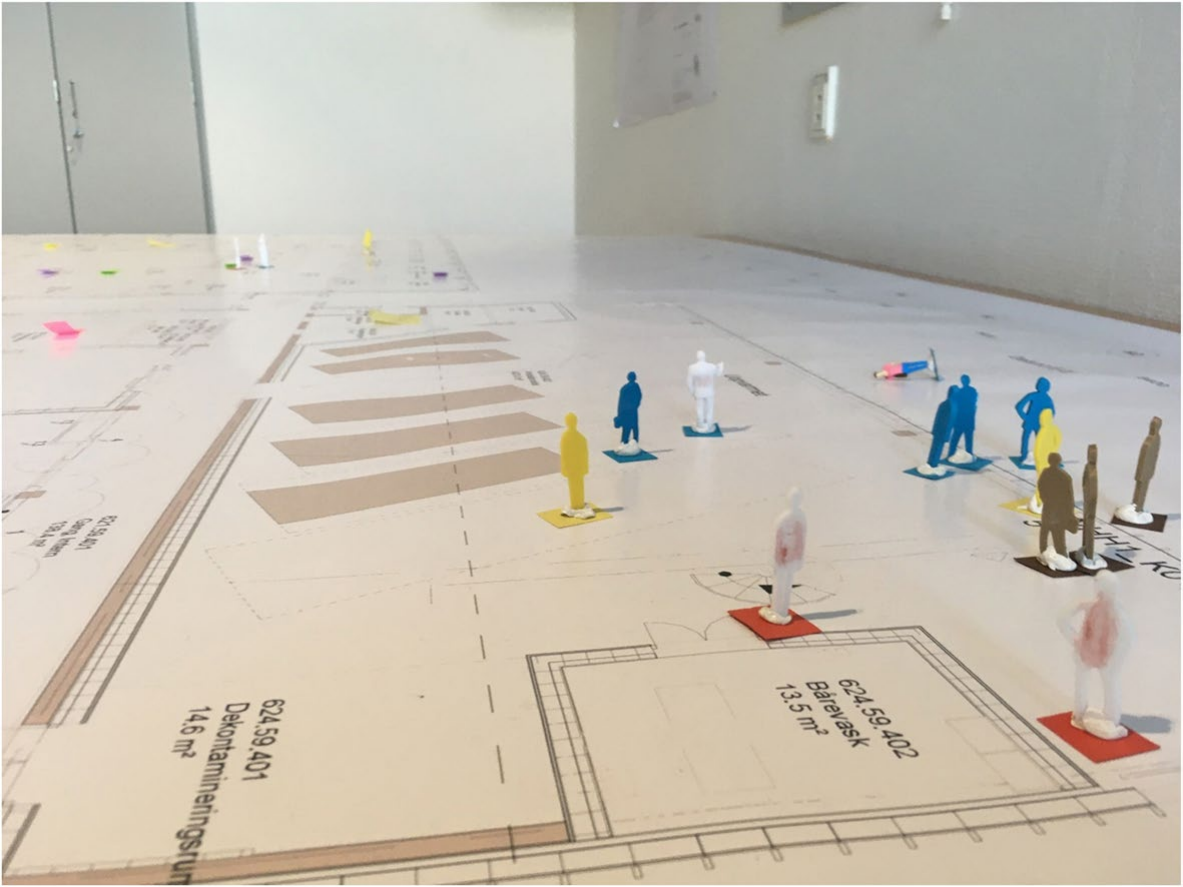
An oilcloth session

The purpose of the oilcloth sessions was to train staff, key employees and managers in new patient pathways and introduce the new physical building design. A key employee is defined as an employee appointed by managers to play a central role in the implementation of a new ED in relation to their department. Oilcloth sessions are a social scientific methodology whereby healthcare managers, representatives of the board of directors, and key employees work together on a blueprint of the layout on A0 paper of the new ED combined with either LEGO figures or other types of plastic figures, generating knowledge, workplace learning and experiences in relation to the implementation of the new ED. The programme theory based on the stipulated plans for the sessions is shown in Table 1 and an oilcloth session is shown in Fig. 1. Thirteen oilcloth sessions were conducted between October 2019 and November 2020. The sessions were carried out in room measuring 30 m^2^ in the hospital. All participants and the facilitator stood around a table to view the blueprint. Each oilcloth session lasted between 1.5 and 2 hours. During the sessions, the researcher, other members of the board of directors, and managers from non-clinical departments were seated on chairs along the walls and were allowed to participate as well. The oilcloth session study forms part of a larger implementation research programme (the FAM implementation programme) initiated in March 2019 and is expected to continue until 2023, when the new ED opens.

**Table 1:** Programme theory for the oilcloth sessions

	If	Then	Intended Outputs	Intended Outcomes
Oilcloth sessions	If the managers and key employees participate in the oilcloth session	Then they will develop their knowledge and learn about the new ED and change their attitude in relation to the new ED	If the managers and key employees change their attitude in relation to the new ED	Then the managers and key employees will respond more positively to the impending implementation of the new ED = increase in acceptability
	If the managers and key employees participate in the oilcloth session	Then they will have the experience of getting involved and get a positive view of the sessions as a useful strategy when implementation a new ED	If the managers have the experience of getting involved and become positive towards oilcloth sessions as a strategy	Then their ownership for participating in oilcloth sessions will increase and they will take responsibility for implementing the new ED = increase in acceptability
	If the managers and key employees participate in the oilcloth session	Then they will gain knowledge and insight into the new physical and material context	If the managers and key employees gain knowledge and insight into the new physical framework	Then their experience of sense of place will increase, which is a key factor for a positive experience towards the new ED and their motivation for supporting the implementation of a new ED = increase in acceptability

### Facilitor and facilitation

Oilcloth sessions were facilitated by two members of the board of directors. One facilitated the two first sessions and another facilitated the remaining 11 sessions. The facilitators were chosen due to their management positions and were therefore expected to have good negotiation, facilitation, and communication skills. In this study, facilitators are understood theoretically as individuals who facilitate the form of participants’ conversations and interactions (a process) rather than the content by making requests and guiding questions that the participants can agree upon [32, 33].

### Participants

The following participants took part in each oilcloth session and interviews: members of the board of directors; managers from the present ED; managers, health professionals, and key employees (e.g., physicians responsible for education) from one or two other specialty departments. The managers were invited to participate by the board of directors and had the responsibility to invite key employees and health professionals from their department. Sometimes, the board of directors also invited managers from non-clinical departments. On average, 15 to 20 participants employed in the departments mentioned in Table 2 were present at each oilcloth session.

**Table 2:** Participating departments

Specialty	Department
Medical specialty	Department of Cardiology Department of Gastroenterology (medical) Department of Infectious Diseases Department of Internal Medicine (including Department of Respiratory Medicine and Department of Endocrinology)
Surgical specialty	Department of Orthopedic Surgery Department of Gastroenterology (surgical)
Emergency specialty	Emergency Department
Other	Department of Clinical Biochemistry Department of Obstetrics and Gynecology Department of Pediatrics and Adolescence Medicine Department of Radiology

The concept of ‘power information’ guided our sampling strategy for the interviews [34]. Items such as the breadth of the study aim, the specificity of experiences, knowledge, or characteristics of the participants affected the sample size [34]. All specialty departments were affected by the implementation of a new ED, therefore the inclusion of participants from all the departments was deemed necessary. Participants had different professions, positions, and represented different medical specialties. This supported the need to explore variation in their experiences and resulted in our choice of inviting everyone who participated in the oilcloth sessions for an interview (*N*=64, including two members of the board of directors). Eleven individuals either did not answer or decline to participate due to heavy workload. Fifty-three semi-structured interviews were conducted due to considerable clinical heterogeneity (Table 3).

**Table 3:** Participants in the interviews

Number (***N***=53)	Profession and Positions
26	Physicians (10 chief physicians, 13 senior physicians and 3 trainee physicians
19	Registered nurses (8 head nurses, 8 charge nurses, 1 assistant charge nurse, 1 clinical nurse specialist and 1 registered nurse
1	Head midwife
2	Managing medical secretaries
2	Bioanalyst (1 bioanalyst and 1 chief bioanalyst)
1	Charge radiographer
2	Members of the board of directors

### Data collection

#### Participant observations and field notes

We used participant observations in the ethnographic study, which allowed us to observe how oilcloth sessions were conducted and to explore contextual factors [35]. Observations were also used to help interpret episodes and situations that participants reported verbally and further understanding of the participants’ experiences [36]. We developed an open observation matrix divided into three columns: (1) observations; (2) reflections; and (3) analytical remarks, which were used at each oilcloth session. At the end of a session, additional notes, reflections, analytical concepts, and remarks were added. Reflections and central points from the field notes were discussed continuously with the rest of the author group.

### Semi-structured interviews

The interviews allowed the researchers to explore how the health professionals, managers, and key employees experienced and evaluated the oilcloth sessions as an implementation strategy. The interviews were conducted between October 2019 and December 2020 by JWK and NTS and were held in the offices of the participants or in meetings room at the hospital. On average, the interviews lasted 39 minutes (from a minimum of 26 minutes to a maximum of 1.03 hours) and covered eight themes contained in a semi-structured interview guide. The interview guide was developed by NTS and JWK and was pilot tested with a senior consultant employed at the management secretariat of the hospital. The interviews were conducted by JWK, and NTS wrote notes and asked follow-up questions. The pilot test led to minor revisions of the interview guide. All 53 interviews were recorded and transcribed verbatim by a research assistant. In total, 790 pages were transcribed. Of the 790 pages, one theme (experiences with oilcloth sessions as a method for implementing a new ED) covered 315 pages which were selected as data relevant for analysis in this study.

### Data coding and analysis

The transcribed data material was read and re-read by JWK to get a sense of the whole dataset. Then, an inductive thematic analysis of the transcribed interview data was conducted [37]. Interview data were condensed in a coding scheme divided into meaning units and then abstracted on a manifest level close to the text and subsequently on a latent level with interpretations using different empirical and theoretical concepts. Finally, sub-themes and themes were created [38]. The emerging themes from the inductive analysis were centred on didactic issues such as objectives, facilitation, and target group. This inspired the authors to structure the themes through a theoretical lens developed by Biggs and Tang [39]. Then, NTS read and discussed the coding scheme with JWK until agreement was reached. This strengthened the validity of the analysis. Finally, the main themes abstracted in the material were discussed with the rest of the author group.

### Conceptual frameworks

In this study several conceptual frameworks are used both in the analysis and in the discussion. In the analysis, Biggs and Tang’s [40] didactic model was used as an overall structural model for our analytical work. In the discussion concepts from Cultural Historical Activity Theory (CHAT) [41] were used, as this theory can explain how tools and signs such as figures, whiteboards, and language mediate and thereby collectively shape and transform the participants in relation to the implementation of a new ED. This also includes the concept of cultural models of Hasse [37], who has defined a network of meaningful connections, e.g. collective thoughts, language, and ways of organizing patient pathways. These cultural models were organized historically over time as general knowledge of the local practice and thereby constitute the local context.

According to Biggs and Tang [39], constructive alignment is central to achieving a high level of learning. The concept consists of two aspects: the constructive aspect and the alignment aspect. The constructive aspect refers to what the learner does, which is to construct meaning through relevant learning activities. The alignment aspect refers to what the teacher does, which is to set up a learning environment that supports the learning activities appropriate for achieving the desired learning outcomes. Key didactic elements should be considered in the organization of a course to secure alignment: the objectives, content, assessment, teaching methods, media, the participants’ prerequisites, and the organizational context [42]. According to Biggs [40], it is important to avoid contradictions and inner tensions between the didactic elements to secure optimal learning. If the didactic elements in the oilcloth sessions are not aligned, participants are forced to deal with tensions.

The interview data were analysed and presented in three phases in the form of preparation, conducting, and execution as well as several didactic elements (in parentheses). The emerging themes were as follows: (a) preparing individually and collectively (teaching method); (b) objectives (objectives); (c) involving participants (the participants’ prerequisites); (d) selecting clinical cases (content); (e) using materials (teaching method); (f) facilitating the sessions (teaching method); (g) temporal structures (teaching method); (h) following up on the sessions (teaching method); (i) adapting to the context (assessment and organizational context). Furthermore, the themes were compared with findings from the ethnographic field study to verify or contrast the findings. This was done by reading the field notes based on specific keywords or meaning content contained in the themes (Table 4).

**Table 4:** Themes and sub-themes

31 Sub-themes	Themes	Biggs and Tang’s (2011)
**Preparation**		
Oilcloth as a method was unknown	Preparing individually and collectively	Teaching method
Material was sent out to the participants before participation by		
The fact that some participants were involved early in the process (10 years ago) has given them a form of preparation that requires that they are not surprised despite the fact that physical spaces have not been as agreed at that time		
Management and preparation are central to a positive experience with oilcloth sessions		
Management representatives are role models and should appear aligned before participating in oilcloth sessions		
Separation between the organizational and the clinical is not perceived constructively (possible)	Conflicting objectives	Objectives
The oilcloth method develops over time (becomes more and more standardized); *strengthens* so that objectives, and purposes become clearer		
Due to the “liquid nature” of oilcloth sessions (experience by participants), a similar and clear objective is required		
Experienced prestige/recognition of being selected by its manager	Involving suitable participants	The participants prerequisites
The target group and the right participants for the new ED (becomes unclear as there is a dual objective with oilcloth sessions; consensus among the managers (management decisions about organization) but professionally close to the practice (the clinics when they are with the patients)		
Relationship between management, habitual ways of acting and the right participants		
Oilcloth made it clear that nursing is not the object of the oilcloth sessions	Selecting suitable clinical cases	Content
The case was copied from the medical record, which became an indicator that it was the medical issue that was in focus at oilcloth sessions		
The case should have been more complex		
Oilcloth sessions were controlled by the physicians		
**Conducting**		
LEGO man a mediating artefact	Using suitable materials	Media
Whiteboard for challenges as a mediating artefact		
The presence of the board of directors was evaluated positively but assumptions were made as to why they were participating [their purpose in participating could have been clarified]	Facilitation the sessions	Teaching method
The presence of the management symbolized that oilcloth sessions are important		
The participants made assumptions about what the board of directors will learn		
Oilcloth sessions became an explicit experience more than practical training	Temporal structures	Teaching method
The introduction went too fast when the participants were new to the oilcloth sessions		
New participants lost track along the way. A visit to the new physical buildings would have provided a different insight		
Time and delay for moving into the new ED		
Time for appointments to be in place		
**Evaluation**		
Experiences about trust/mistrust of the implementation of the new ED depending on management and preparation in the oilcloth sessions	Follow-up on the sessions	Assessment
Missing plan for follow-up (consequence of an experience that oilcloth sessions are a detached and sporadic activity). The consequence can be uncertainty about the new ED		
Uncertainty about follow-up and responsibilities		
Experiences with previous collaborations in daily practice affect the experience of oilcloth sessions [positive and negative experiences]	Adapting to the context	Assessment and organizational context
Oilcloth sessions can be experienced as a game but also as a strategy for implementing the future		
Waste of public funds		

## Results

The analysis showed that experiences of the health professionals, managers and other key employees with oilcloth sessions as an implementation strategy were based on an interplay of different actors, temporal structures, and a sensitivity to the context that can be described in terms of the three temporal sequences and nine main themes (a–i): preparation phase: (a) preparing individually and collectively, (b) objectives, (c) involving suitable participants, (d) selecting clinical cases; execution phase: (e) using materials, (f) facilitating the sessions, (g) temporal structures; evaluation: (h) following up on the sessions, (i) adapting to the context. The themes included 31 sub-themes illustrating the complexity of experience using oilcloth as an implementation strategy when implementing an organizational structure as complex as an ED (Table 4). The results are presented according to the three phases and nine main themes.

### Preparation phase

#### Theme (a): preparing individually and collectively

According to Biggs, quality learning takes time in preparation. Participants in this study highlighted preparation as a key element. Preparation took place on an individual level; some participants experienced it as appreciation to be selected by their managers to participate in the oilcloth sessions:

I think it's cool that [the chief physician] asked and invited me. I think he sent a PowerPoint presentation about how it [the oilcloth session] would be conducted and what the objectives were, so I could prepare myself […] (a representative from the medical specialty).

Being asked to participate and receiving written material was appreciated, and it enabled the participants to prepare for the oilcloth sessions (regarding time, place, and duration). Some participants mentioned that they did not receive the material from their managers before participating in the session.

Preparation also affected a collective level, including participants’ compliance with agreements established before the oilcloth sessions (e.g., those between the board of directors and the managers from the ED or specialty departments). Agreements could be about the overall objectives or direction of the future ED. However, several of the participants did not experience that the managers were aligned before the sessions. Alignment can be understood as a collective way of thinking, understanding, and interpreting the rules and direction of the new ED. One participant expressed this:

I think the oilcloth sessions require that the managers are aligned. It was not my experience at the beginning of the oilcloth sessions. We participated in some oilcloth sessions where we actually discussed things that we should have agreed on [beforehand] at management level (a representative from other departments).

After conducting the first two sessions, this topic was discussed by some of the managers. They agreed that they should appear as role models for their employees and not create unnecessary worries among the employees by not appearing aligned. Against this background, the board of directors introduced pre-meetings with managers from the ED and/or specialty departments. These pre-meetings became a learning space for the managers, with the purpose of creating “collective meaning and thinking” [43] that could lead to a collective consciousness between the managers in relation to the new ED, before participating in the sessions.

#### Theme (b): objectives

The objective of the oilcloth sessions seemed to be unclear to many of the participants, causing some confusion among them. The field study showed that several different objectives and outcomes were presented in the sessions. A stated objective was: “We are going to focus on the organizational and not the clinical work” (field notes, oilcloth session no. 1). In this context, the “organizational” was expressed as new ways of working together for all health professionals in the new ED, including new ways of organizing the work of the specialist physicians. Another stated objective was: “We should work with the current patient pathways and focus on what bumps we can improve clinically” (field notes, oilcloth session no. 2) and yet another objective that was stated was: “We must work with future optimized patient pathways” (field notes, oilcloth session no. 3). According to Biggs and Tang, objectives exist to provide a focused mindset for the participants. When the objectives were stated in a continuum between the professional and organizational and in relation to the current or future patient pathways, the consequence was an unfocused mindset among the participants:

And then, of course, there will be time wasted on debating some clinical things, which may be fundamentally unimportant in relation to the organizational context (a representative from the surgery specialty).

Review of existing patient pathways at the oilcloth session triggered a positive “aha experience” for several of the participants because it clarified how other specialties handled the patient pathways. This internal identification process must be recognized by outsiders for an objectified collective identity to emerge. As one participant explained:

In our [surgical] specialty, we receive patients according to slightly different procedures than you do [referring to the managers from the existing ED]. Both physicians and nurses are physically located in the same room, so we jointly discuss the patient. It is characteristic of us, and I think we should continue with this organization in the new ED (a representative from the surgery specialty, fieldnotes, oil cloth session no. 2).

Such a narrative of the surgical practice was a source of motivation among the surgical participants to convince the participants and managers from the ED that tasks should be performed in line with the surgical identity and practice when working with existing patient pathways. After this statement in the session, the dialogue focused on physical spaces and the possibilities and limitations the participants considered for a future collaboration between nurses and physicians. In this way, the participants came to know each other, themselves, and the world while jointly enacting collective practices mediated by cultural tools (language as a symbolic system), building on efforts of each other and on achievements of previous generations (the “we”), and cumulatively expanding on and amplifying these practices through the oilcloth sessions.

Several of the participating managers disagreed that the objectives were to work with future clinical patient pathways or the organizational structure, which gave rise to frustrations among some of the key employees. These different foci of the objectives were conveyed in discussions that either became very detailed regarding the clinical practice: “Should we take blood samples for both electrolyte counts and C-reactive proteins” (field notes, oilcloth session no.7) or regarding the organizational “In the new ED, it will be the flow master who ensures the flow on both floors” (field notes, oilcloth session no. 7). The different foci caused a lot of discussions in the first sessions:

We spent so much time discussing whether it was the organizational or the professional clinical that was at the centre. It was a waste of time (a representative of the medical specialty).

The consequence of the perceived lack of clarity of the objectives of the oilcloth sessions was that the participants perceived them as fictitious and left the sessions with the experience of creating a them/us situation that they perceived as group boundaries, giving the feeling of exclusion and that no result was generated from the sessions; instead, they were regarded as a waste of time and resources:

I think it is impressive how many people you can gather… for so little. […] What did we learn from it? I would like to see a sign hanging on the door stating how much money we have spent. Some, maybe the managers, probably learn something but we clinicians, we did not learn anything (a representative from the surgery specialty).

Even so, time became a key factor in consolidating a common objective with the oilcloth sessions. Over time, the sessions became more and more standardized and objectives “solidified” and became clearer and more distinct to the participants, which was perceived positively. One participant said:

I think the challenge at the beginning of the oilcloth sessions was ‘what were the objectives exactly?’ […]. I do not think there was anyone who knew, either among the board of directors or from either of the participating groups. Therefore, I think we quickly grabbed a case we knew and worked with that. Today, I am convinced that we must develop new patient pathways, and that this will contribute with an increased focus for the participants (a representative from the emergency department).

The clarification of the objectives created focus for the participants over time, which was evaluated as positive and that the oilcloth sessions were a useful strategy in relation to the implementation of a new ED.

#### Theme (c): involving participants

When designing implementation processes through oilcloth sessions, the question of who should participate is extremely relevant [13]. The participants also linked this question to conflicting objectives for the oilcloth sessions. As one participant stated:

As the objectives of the oilcloth session were unclear to us, the appropriate or right participants were not invited (a representative of the medical specialty).

If the objective was to work with the organizational aspect of implementing the new ED, some managers considered clinical development nurses and physicians responsible for education as the right target group to be involved. If the objectives were to work with the clinical aspects in the form of present or future patient pathways, clinicians were assessed as the right target group. One manager expressed:

Several physicians expect to be invited to the oilcloth sessions because they are the ones who take care of the patients (a representative from the medical specialty).

The lack of clinicians was perceived as a didactic error in the oilcloth sessions. As one participant explained: “Clinicians who handle the clinical work should have been represented to give realism to the oilcloth sessions” (a representative from the emergency department). The lack of realism was mentioned by several of the participants. “Constructive inputs require that you have been in an ED before and know how it works” (a representative from the surgery specialty).

At a later time point in the oilcloth sessions, trainee physicians were invited to counteract this issue, which was evaluated positively by many of the participants. One representative from the medical specialty stated:

It was clear that the trainee physicians voiced their concerns from their position close to the clinic, which are different from the concerns of, for example, the top managers.

In addition, the question was raised whether there were other target groups that should have been involved, such as porters or the pre-hospital emergency services. As one participant expressed:

The patient pathways start with the patient being picked up by the ambulance, therefore they should have participated in the oilcloth sessions (a representative member of the emergency department).

However, a member of the board of directors stated the importance of being in control in the internal organization before inviting external partners, a statement that was related to the objectives of the sessions.

Some participants (e.g., specialist development nurses) assessed that, in the future, they would only have sparse contact with the new ED and therefore they felt out of place at the oilcloth sessions. During the interviews, it became clear that several of the management had selected key employees to participate because they were perceived as good role models in their departments and therefore were expected to speak positively about the future ED. Other arguments were that they were good advocates for the new ED, had extensive professional knowledge or had a good strategic mindset. This was deemed necessary by managers to avoid unnecessary concerns and barriers in relation to the reorganization and implementation of the new ED.

#### Theme d: selecting clinical cases

Clinical cases referring to existing patient pathways was mentioned by the participants as central for learning. A case was a patient pathway retrieved from a medical record. The uses of these cases gave the participants insight into how the patient was handled by other professions or elsewhere in the organization and became a way of avoiding fragmentation between specialties and a way for the participants to learn across social and cultural practices.

Before the oilcloth session took place, the details of two cases were distributed to the participants. The practicalities of selecting and distributing the cases from the managers to the selected participants was experienced as working well. The selection of the cases was central to the participants. The cases should not be too simple; they should be examples of worst cases because the participants experienced these as resource demanding both physically and mentally in their daily practice. A representative from the surgery specialty stated:

As we say, it is very easy to receive the patients if we have physicians available […] But what do we do when we [the physicians] are not available, or when there are three very ill patients at once? I think it would be good to go a little further with these types of cases. As realistic cases as possible.

The cases included were often considered too medical by participants from professions other than physicians, because the cases focused on treatment only. Consequently, some of these participants perceived oilcloth sessions as constructed and controlled by the physicians with a medical background. One of the participants explained: “… and the details are copied from the medical record and that was very much the case” (a representative from the surgery specialty). The fact that the details were copied from the medical record and not from the nursing record led to other professions felt excluded and the cases were perceived as too simplistic by only focusing on the medical professions. A representative from the medical specialty stated:

We felt that it was very medically orientated, and we felt a little excluded; the cases were very simplified and hierarchical.

Considering these medical cases as cultural tools and signs, they suggest that the oilcloth sessions were experienced as a method that supported hierarchies whereby physicians and treatment became the dominant focus in contrast to nursing care or therapeutic treatment (e.g., mobility or rehabilitation plans).

### Execution phase

#### Theme e: using materials

Two central artefacts were highlighted by several of the participants; the small plastic figures (named “LEGO men” by the participants) and the whiteboard introduced during the oilcloth sessions. Although on the one hand several of the participants found them a bit comical, the figures were found useful:

And even though it seemed a little … here we are playing with small figures it was … I think it was very rewarding (a representative from other departments).

The figures were compared with LEGO men because several of the participants had previous experience working with improvement processes in the form of LEAN [44], where LEGO figures were a regular component. The LEGO figures became common tools that the participants used to understand and mediate their experience and understanding of the implementation of the ED.

The figures were designed differently depending on which physicians the figures were to symbolize (the full figure was an experienced physician and trainee physicians were symbolized by a narrow figure). These different depictions triggered many comments, laughter and created a positive climate among the participants. A participant stated:

It is instructive for me to hear and see why the other medical specialties think it is a trainee physician they believe should receive the patient [shown by moving the figure on the oilcloth]. It tells me something about their collective thinking and organization within the specialty (a representative from the medical specialty and field notes, oilcloth session no. 10).

In this way, the use of the figures became a sign of collective ways of thinking and acting, depending on which figure the participants jointly agreed should be used.

Another mediating tool that was evaluated positively was a whiteboard that was introduced along the way. As mentioned earlier, several of the participants did not consider it appropriate for managers to have a discussion (sometimes in a sharp tone) in front of their selected key employees, because this could create fear and concern among employees, which could ultimately complicate the implementation of the new ED. By writing down and placing disagreements and ambiguities on the board, the board functioned as a mediating tool by collating different understandings of different points of view but also as a sign of differences in cultural practices within different specialties and professional identities. A participant stated:

Something I think is good about the oilcloth sessions is that when we encounter a challenge, we park it [on the whiteboard] and then we work with it later, instead of discussing loudly in the room (a representative from the emergency department).

#### Theme f: facilitating the sessions

Facilitation was an integral part of the oilcloth sessions and it involved many of the participants. The oilcloth sessions were facilitated by a deputy managing director and the representation of managers and the CEO participating in the room. The presence of members of the board of directors was evaluated differently. Some participants wondered about the role that, for example, one of the representatives from the board of directors took on:

Then one of the representatives from the board of directors sat and wrote post-it notes. Why? I have no idea. Any other participant or consultant could have done so (a representative from the medical specialty).

Others found it positive that the members of the board of directors were represented, because it signalled the importance of conducting and participating in oilcloth sessions. One representative from the surgery specialty expressed:

It is an important signal and of value to everyone in the room that the members of the board of directors prioritize participating in the oilcloth sessions. The new ED is thereby made an important and prioritized change in the organization.

But the participation and facilitation of the oilcloth sessions by the board of directors was also questioned with reference to hierarchies and power: “If you want to hear the truth from the managers, you need impartial facilitators” (a representative from the surgery specialty). Through the interviews with the managers and key employees, it became clear that members of the board of directors were assigned a dominant position, which was assessed as hampering communication and the process of the oilcloth sessions, especially among participants who considered themselves less significant and with less influence than, for example, many of the managers. A participant mentioned:

I always considered what I said because the managers were in the room and I did not want to appear ignorant (a representative from the surgery specialty).

Although the managers had invited relevant key employees and health professionals from their department, expectations of hierarchies were brought into the oilcloth sessions and became a guiding and mediating force for some of the participants and affected how they participated and communicated during the sessions.

Another consideration concerned differences in the professional identity of those who facilitated the oilcloth sessions. As mentioned earlier, many of the participants expressed that the oilcloth sessions focused too much on the medical profession. This was reinforced by the observation that the deputy managing director who facilitated the sessions was a physician and placed himself at the table close to the other physicians and top managers. Conversely, it was a strategic choice from the board of directors because this deputy managing director was newly appointed. A member of the board of directors expressed:

It is a good opportunity to learn about the organization and find out what is going on in the medical specialties and with the specialists in relation to the forthcoming reorganization.

Participants who joined some of the last oilcloth sessions for the first time expressed that facilitation was lacking. They felt that the introduction to the sessions went too quickly, was too superficial and not empathic. As one participant said:

You can feel that several of the participants had participated many times. Newcomers could not keep up at all when introduced (a representative from the medical specialty).

Several new participants pointed out that they lost track along the way due to a lack of management and facilitation, thereby highlighting the importance of the facilitator’s role, competencies, and experiences. Many participants said that a visit to the new physical buildings before attending the oilcloth sessions had given them a different insight into the new ED and an opportunity to better keep up in the sessions.

#### Theme g: temporal structures

Many of the participants had opinions about time and timing. Patterns of time can be viewed either as an objective entity (the metaphoric clock), which is mechanical, linear, and quantitative [45], or as a subjective conceptualization that is qualitative, discontinuous, and cannot be presented in neat, easy-to-measure and synchronized formats [46]. During the oilcloth sessions, time manifested itself through three temporal sub-themes: having sufficient time for the sessions, delayed deadlines and planning the timing of the sessions.

Sufficient time for the sessions was discussed in the form of the duration of the oilcloth sessions. Each session took 1.5 to 2 hours and most specialties participated in the sessions one or two times. Only the managers and the key employees from the existing ED participated in all 13 oilcloth sessions, because they were to be the top managers in the new ED. Most participants rated the duration and the period between the sessions as appropriate. This contrasted with others who found the timing for the oilcloth sessions inappropriate due to delayed deadlines for moving into the new ED. One participant said:

In theory, it is probably very smart, but I think we are a little too early. Because we do not have proper agreements in place about what the staff should do where and how (a representative from the surgery specialty).

For some participants, the timing of the oilcloth sessions created tension between the need for better temporal coordination and the organizational circumstances. Several of the issues discussed in the oilcloth sessions were already stated in written documents and agreed by the board of directors and the managers from the ED but were not being followed strictly by the managers of the specialty departments and therefore were subject to ongoing debate.

Timing of the oilcloth sessions was often mentioned by the participants. The timing was deemed inappropriate because within some specialties and professions, there was opposition to the establishment of the new ED. This resistance was expressed during the oilcloth sessions. A participant mentioned:

But it is because they do not think that the new ED is important yet and others are generally opposed to the reorganization that is happening. It became clear at the sessions (a representative from the other department).

Other participants experienced that the oilcloth sessions mentally burdened them in their daily practice as one participant said:

Why now? [with reference to conducting the oilcloth sessions]. There is plenty a time until we are going to move and there are other tasks that we have to take care of that are more important. It becomes a stress factor (a representative from the surgery specialty).

Several of the participants expressed that the oilcloth sessions were a burden, but this experience probably related to the contextual situation they found themselves in (e.g., the closure of a department). One participant expressed:

For us it is not just about participating an oilcloth session. It is a sign of the major reorganization we are about to undertake, the closure of a section and the merging of our staff (a representative from the surgery specialty, field notes, oilcloth session no. 3).

Others approached the oilcloth sessions with a more positive attitude and saw them as an opportunity to work with the reorganization and implementation of the new ED based on the knowledge and experience achieved during the sessions regarding future collaboration with other specialties. One participant explained:

Yes, it is mentally time-consuming, but it also gives me the opportunity to think further, plan what the next step should be, depending on what we experienced at the last oilcloth sessions (a representative from the other department, field notes, oilcloth session no. 13).

Thus, temporalities such as timing and time both as an objective entity and as a subjective conceptualization played a central role in the participants’ experiences of securing alignment in the oilcloth sessions.

### Evaluation

#### Theme h: following up on the sessions

According to Biggs and Tang, the connection between in-class and out-of-class is important to ensure optimal learning. After the oilcloth sessions ended, most of the participants stated that they lacked a follow-up plan. Some managers had been given tasks to continue with on the future reorganization, but most of the participants had no idea what was going to happen after the sessions. They did not know if they should participate in more sessions, if there was an overall announcement or feedback from the oilcloth sessions or if there was something they had to follow up on. One representative from the other another department expressed: “I also do not know what the plan is after the oilcloth sessions” Several participants initially acknowledged the invitation from the board of directors to participate in the oilcloth sessions and perceived it as an opportunity for involvement. A key employee said:

I think it is a gesture from the management to invite us, to involve us. I think this is a strong signal to send to us in the other medical specialties (a representative from the other department).

The lack of a plan for the next steps in relation to the implementation plan created doubts about the board’s actual intention and willingness to involve the specialties:

I am not sure what this circus was. Did they [the board of directors] really want our ideas and feedback or was it just a superficial action, now that we do not hear anything more about what is going to happen; so they [the board of directors] could say that we were involved? The question is whether there is control of the implementation of the new ED? ” (a representative from the medical specialty).

The lack of feedback and a follow-up plan created distrust about the agenda and whether there was control over the implementation of the new ED. The oilcloth sessions were perceived as a detached and sporadic strategy that ultimately created uncertainty about the future implementation of the new ED:

Yes, it can be characterized as a somewhat random activity we have participated in. I do not know what significance it has for the future implementation of the new ED (a representative from the other department).

#### Theme i: adapting to the context

The participants’ general evaluation of the oilcloth sessions was linked to the historical and situational context to which the individual specialties and participants relate. Contextual issues in the form of historical practice emerged when asking for a general evaluation of the oilcloth sessions as a strategy for implementation of a new ED. As one representative from the surgery specialty explained: “Oilcloth sessions were taken seriously due to ownership of the specialized patients, who are on loan in the new ED.” This statement was an example of a historically manifest specialized “silo thinking” that the political objectives of the establishment of new EDs were meant to overcome. Other participants experienced the oilcloth sessions as a means to an end, namely to ensure that specialty patients were offered the most efficient and quality-assured specialist-related patient pathways when they were admitted to the new ED and before they were either discharged or transferred to the specialty departments. A key employee stated:

Oilcloth sessions can be experienced not only as a game but also as a strategy for implementing the future, including collaboration and organization with other medical specialties (a representative from the emergency department).

Other participants evaluated the oilcloth sessions in relation to the situational context as: “The oilcloth sessions were assessed as a waste of money and a waste of public funds in proportion to the results of the sessions” (a representative from the medical specialty)*.* This statement needs to be viewed in light of the fact that the participant came from a specialty department that was to have only a few beds in the new ED. Several of the participants from this department shared their experience:

There was not much room for being heard. It seemed like an agenda had been set before we got to the oilcloth sessions, an agenda that left no room for discussion (a representative from the medical specialty).

One of the specialty departments had to close their ED and merge with the new ED. In connection with this, the use of oilcloth sessions was compared with Lars von Trier's movie film “Dogville”. The comparison could be interpreted as arriving at oilcloth sessions [the new ED] and being granted asylum [their own emergency department had to closed down] in return for work [in the future the specialists must be on call in the new ED]. The participants’ experiences of the usefulness of the oilcloth sessions were thus shaped by the “fate” they thought their department would have in the new ED. It became clear in an interview with one deputy manager who stated:” we thought we knew of all the challenges”, that these historical and contextual thoughts must be learned by the managers and the facilitator to make it possible to act on the participants’ experiences in relation to oilcloth sessions as an implementation strategy.

## Discussion

The aim of this study was to explore challenges with oilcloth sessions as an implementation strategy based on the experiences of healthcare professionals, managers, and other key employees who participated in the sessions. Through the perspectives of Biggs and Tang’s didactic model [39], nine themes emerged attributable to three temporal phases: the preparation phase: (a) preparing individually and collectively, (b) objectives, (c) involving participants, (d) selecting clinical cases; the execution phase: (e) using materials, (f) facilitating the sessions, (g) temporal structures; the evaluation phase: (h) following up on the sessions, (i) adapting to the context. The challenges are discussed in relation to Biggs and Tang's theory of constructed alignment, which is a principle for designing learning activities that address the intended learning outcomes. Also, some themes are further discussed using concepts from Cultural Historical Activity Theory (CHAT) [47], which explain how tools and signs such as figures, whiteboards, and language mediate and thereby collectively shape and transform the participants in relation to the implementation of a new ED.

Applying Biggs and Tang model [39], the managers and key employees are the learners and the learning environment is the oilcloth sessions as an implementation strategy set up and facilitated by the board of directors to support the implementation of a new ED. If the intended learning outcomes was fulfilled, then the participants experience the sessions positively, which was expected to increase participations in oilcloth session and increase responsibility and motivation for supporting the implementation of a new ED according to the programme theory (Table 1). The results showed that, most of the time, the didactic element in the oilcloth session was not aligned. The consequence was that the participants experienced different tensions and ambiguous experiences with oilcloth sessions as an implementation strategy. These experiences created barriers to positive responses, increased responsibility, and motivation for supporting the implementation of the new ED.

### Preparation phase

An important challenge in the preparation phase was the objectives, which were stated in a continuum between the professional and organizational and in relation to the current or future patient pathways. The consequence was frustration and an experience of wasted time and effort as well as an unfocused mindset among the participants. Ordóñez and colleagues [48] argued that defining the objectives has been promoted as a halcyon pill for improving employee motivation and performance in organizations and specific objectives boost performance. When focusing on existing patient pathways, a positive “aha experience” was triggered because it clarified how other specialties handled the patient pathways. From the perspective of CHAT [41], this experience can be understood as a collective identity whereby different specialties and/or professions differentiate themselves from others by drawing on criteria of community, establishing a “we” and a sense of reward within their subgroup.

In this study, the participants’ frustrations were related to conflicting objectives caused by participants being prone to concentrate on only one objective, e.g. the clinical or the organizational. Objectives that are easier to achieve are often given more attention [49], and this was also the case during the oilcloth sessions; especially trainee physicians gave more attention to the clinical objectives than the organizational objectives. Furthermore, research has shown that unclear or even contradictory objectives can lead to unsuccessful implementation [50], tasks not being performed at all, and the creation of a learning environment where the participants cannot be at their most effective [39]. The results show that this was the case for some of the participants who did not have positive experiences with the oilcloth sessions as an implementation strategy because of conflicting objectives.

Another important consequence of the conflicting objectives related to the selection of cases and participants. When the focus was on existing patient pathways that was experienced positively by clinicians, e.g. trainee physicians. Thereby alignment was achieved between the objectives, target group and the cases. In these examples, the participants learned across social and cultural practices [51]. The concept of boundary objects, i.e. objects that are part of multiple social worlds and facilitate communication between them [52], is relevant in this context. When cases, objectives and suitable participants were aligned, it created curiosity, and communication and motivation among different participants and managers from different specialties were created. From this perspective, the oilcloth sessions could be seen as a boundary object that could potentially bridge gaps between specialties and allow them to work together without consensus. This became evident in the interviews, where many participants and managers expressed that they had experienced these sessions positively and found them significant for a positive response to and increased responsibility in relation to the implementation of a new ED.

Conversely, cases with a focus on future patient pathways, and thereby the organizational issues, created many contradictions and tensions for some of the participants and managers. One reason was that some of the organizational issues relating to the new ED were still being negotiated with the board of directors, e.g. the number of specialists who were to be present in the new ED. This meant that the participants constantly negotiated their mutual working relationship, which often proved to be ambiguous and frustrating and meaningless to the participants. Due to the organizational ambiguities and based on the way the hospital was formally organized, dialogues arose between the ED and the specialty departments. Nugus [53] referred to these as dialogues between hierarchically specialized departments, where the distinction between organs determines whether or how physicians should be involved in an ED patient pathway. The consequence was that the organizational as an objective became too difficult to work with and fulfil as an objective in the oilcloth sessions and it created boundaries and hindered collaboration across specialties.

The difficulties became evident through positions of power that were expressed explicitly through a narrative of “us and them”, which predominantly related to an interdepartmental hierarchy of specialized departments dealing with particular body organs, versus emergency medicine, which is a generalized specialty [53]. A positional hierarchy of formal, hospital-wide positions of individual seniority was overruled by the interdepartmental hierarchy when disagreement arose [53]. Hierarchies and power can be characterized as Organizational Implementation Context factors, which are factors likely to influence implementation processes. Dhillion [54] argues for the importance of managers being aware of power relations and various ways of manifesting power to secure a good position when implementing organizational change. Based on the results, the author group recommend that key organizational issues must be decided, e.g. the number of specialists, if oilcloth sessions as an implementation strategy are used as a strategy focusing on the objectives of future patient pathways. This recommendation must be seen in the light of the hospital as an organization where hierarchies and power are significant contextual factors, and managers and facilitators must be prepared for and handle in a positive way. Hierarchies and power continually mediate relationships and moment-to-moment interactions in the oilcloth sessions as a way to negotiate order [55]. If this is not handled in a positive way, these factors can lead to negative experiences of oilcloth sessions as a strategy with the consequence of increased complexity regarding the implementation of the new ED.

The study showed that the participants experienced the learning objective of the oilcloth sessions as performance goals that led to competition between the specialties and between different professions. Scholars have previously pointed out that learning objectives should be used in complex situations rather than performance goals, because they support collaboration and learning [56]. The board of directors did not intend for the objectives of the oilcloth sessions to be performance goals and competitive, but this became a side effect of lack of alignment between objectives, suitable cases, and participants.

### Execution

In the execution phase, the use of LEGO figures functioned as mediating tools and signs that supported changes in the external environment by making future tasks visible and distributed among relevant managers and in the communication between the managers [47]. These tools and signs also supported change in the internal psychological and social processes of the participants, purposefully creating a common understanding that could support a successful experience of participation in oilcloth sessions [41]. The study shows that it is through these artefacts that cultural practices come into being and are perpetuated [57]. Materiality and artefacts are important mediators when we organize learning and plan implementation processes, and more implementation research focusing on the importance of the mediating effect of materiality in implementation processes is needed.

Implementation facilitation (IF), an important and often discussed implementation strategy [58, 59], has proven successful in supporting implementation innovation efforts and contributing to learning capacity [60]. IF is defined as both doing and enabling, e.g. a facilitator may provide education or monitor uptake of the innovation. In this study, the facilitation has some similarities with IF [61], e.g. by enabling dialogue between the participants and progress in the oilcloth session but not by doing and planning the entire implementation process. The literature underscores the characteristics of a good facilitator, e.g. the ability to empathize and understand the needs of others and to know when to speak [62], and in particular skills such as too continuously monitor and adapt implementation as well as operationalizing and detailing the implementation strategies [59, 63]. Even though the participation of the board of directors was evaluated positively by many of the top managers, three interrelated barriers regarding the facilitation of the oilcloth sessions were highlighted: hierarchies and power, professions, and competencies.

The choice of two of the board of directors as facilitators was a strategic management decision, and the oilcloth session was thus viewed as a good opportunity for them to learn about the organization. The literature recommends that a facilitator is selected based on the person’s facilitation skills [61, 62, 64]. However, several of the participants who attended the last oilcloth sessions did not experience the facilitation as skillful. They believed the facilitator lacked empathy or a willingness to understand their needs; the introductions went too fast and were too superficial. From a CHAT perspective [41], understanding the concept of “oilcloth sessions” is significant for the participants’ ability to develop abstract thinking in relation to the new ED. If the introduction of the oilcloth concept is done too superficially or too quickly, the possibility of internalizing both a professional or organizational understanding of a future ED decreases, which increases the risk that a new ED makes less sense to the participants and creates resistance to implementing a new ED. Participants with experience of lack of facilitation asked for a visit in the new physical buildings before attending the sessions, because it could help them in the execution of the sessions.

The position of the deputy managing director created other barriers for some of the participants because they believed his presence could hinder honesty and communication due to unequal power relations. Being a physician or a nurse had significance for the facilitation due to different professional identities and thereby different foci. On the one hand, this became a barrier to motivation for participation for some participants with a different professional background. For other participants, the presence of a physician as facilitator created credibility and became a sign of the importance of these oilcloth sessions as a method to support the implementation of a new ED. As facilitation of implementation processes is complex, it is easy for facilitators to become overwhelmed, and it is not only recommended but a prerequisite that facilitators have the right skills or seek training, mentoring or consultation before entering implementation processes. Scholars have been concerned about defining good facilitation skills [61, 63, 64], but the importance of position and profession is less explored and needs to be examined further. Our results highlight that positions and professions can be both facilitators and barriers for experiences of alignment, depending on the context in which the facilitation is situated.

### Evaluation phase

The connection between in-class and out-of-class is a facilitator for ensuring alignment [39]. Lack of information and follow-up plans created uncertainty about the intentions of the oilcloth sessions and led to an experience of shallow involvement. The consequence of not knowing what the further plans are after participating in the oilcloth sessions resulted in participants expressing distrust about the process and the board of directors and questioning whether there was any control over the implementation process. The invitation to participate in oilcloth sessions can be seen as what Hasse [65] defines as a cultural marker; in this case, a strong board of directors that wants the involvement of the organization's members. However, the lack of information about the further implementation process was perceived as “pretended involvement” or a toxic involvement as described by Oliver and colleagues [66] and was considered less credible. Oilcloth sessions were experienced as a disconnected, isolated strategy in the overall understanding of the implementation process, which constituted challenges to achieving the objective.

Consideration of alignment is also highlighted as important in implementation research and especially within healthcare organizations that are characterized by multiple objectives and power relations that challenge the implementation [67]. Alignment is discussed in implementation science (IS) around two complementary dimensions: structural and social [68, 69]. Both dimensions were central in this study; structural dimensions, e.g. lack of communication about a clear plan, became a barrier for alignment, whereas a social dimension, e.g. pre-meeting with managers before participating in the session, created a common understanding supporting alignment. Based on the results, it is recommended that a clear plan is communicated from the beginning of the session, and that a follow-up plan for further work after the sessions is distributed so that the connection between the oilcloth sessions and the overall implementation process becomes clear to all participants. The results from this study can contribute to the alignment literature in IS. Further research is required in relation to this topic [67] and the study contributes further with knowledge of alignment in relation to a particular implementation strategy.

Finally, the participants’ evaluation of the oilcloth sessions was linked to contextual issues that extended beyond the common definitions of context in the IS literature understood as the environment in which a concrete change is to take place [70–72]*.* Both historical and situational contextual issues were raised by the participants, and these were shaped by the fate they believed their department would have in the new ED. This notion of future fate forms part of cultural models [37]. As an example of a cultural model, some participants evaluated the sessions in relation to securing the pathways for the specialized patients who would only stay temporarily in the new ED. Participants from one of the surgical specialty departments evaluated the session in more situational terms as a strategy that was a waste of time and money, because the implementation of the new ED was not perceived as a huge change for them. These differences in perspectives also defined the context and were thus not contextual factors that could be known or described at the outset of the oilcloth sessions or a study but must be learned over time. Oilcloth sessions have the potential to be adapted with clear objectives and suitable participants, cases, and materials, but only with the recognition that the context is important for ascertaining appropriate alignment.

### Strengths and limitations

Educational strategies are widely used to initiate and implement change in healthcare [73]. In this study, oilcloth sessions were used in the initial phase of the implementation process focusing on the initial considerations regarding the host setting [74]. A strength of this study is the focus on the analytical characteristics of the oilcloth sessions as an implementation strategy, e.g. objectives and suitable participants. This contrasts with the Cochrane Effective Practice and Organisation of Care Group [75], which focuses primarily on the mode of delivery of educational strategies. Our results concerning the temporal aspects of the sessions show that an oilcloth session is becoming as a strategy and therefore solidifying in format to become a standardized implementation strategy that can be used in other phases, e.g. during the active implementation phase or in an evaluation phase to follow up on already established patient pathways.

Another strength of the study is the use of two learning theories: Biggs and Tang [39] and CHAT [76], something that increases the validity of the results [77]. Many learning theories concern individuals’ learning [78], which is reflected in the use of educational implementation strategies because they typically focus on individuals’ capacity and behaviour change [23]. In contrast, CHAT as a learning theory concerns the social and collective through mediation of tools and signs and thereby complements both individual learning theories and IS by focusing on the collective and materialities, which may provide important insights in relation to implementation processes.

Finally, a strength of the study is the combination of participant observations and interviews. This has made it possible not only to talk to the participants about their experiences with participating in oilcloth sessions but also to learn directly from the participants by attending the sessions. This engagement has the advantage of yielding highly detailed and in-depth data, and it has enabled relationships to be built with participants and access to insider perspectives [79, 80]. Being physically present provides an opportunity to learn about less obvious contextual factors, experience power relations in the room and the mediating but often tacit functions of materialities. Interviews alone would not have made it possible to obtain many of these insights. Gertner et al. [81] advocate using ethnographic approaches in IS because they believe these methods are particularly well suited to capture contextual factors and to incorporate the perspectives of end users in implementation processes. By combining the methods, the reliability of the results is enhanced by cross-checking information and returning to the same topic in different circumstances and by comparing statements with observations [77].

One limitation that could be discussed is the position of the researchers. The insights that researchers can get from the participants’ learning processes at the oilcloth sessions depend on their position and access to the field [37]. Formal access to observe the oilcloth sessions was given by the board of directors. This meant that JWK or NTS often arrived earlier at the oilcloth session together with the board of directors and top managers from the ED. Further, they attended pre-meetings and were given the opportunity to stay in the room after the oilcloth session when the board of directors and the top managers evaluated the session. Although JWK or NTS presented themselves as researchers at every oilcloth session and briefly explained the purpose of the study, it became clear that their position was not recognized by all participants. In the subsequent interviews, several of the informants positioned JWK and NTS with the board of directors and in a social role of being closer to the “strong power”, understood as a research consultant [38] when they asked questions such as: “Who initiated this study?” and “Has the board of directors commissioned this study?”. By clarifying for the participants that the study was independent of the board of directors and other top managers, it made them willing to share additional perspectives.

Another limitation of the study was that we did not succeed in clarifying to all participants that the project was not initiated by the board of directors. This oversight may have affected the data collection; some participants became reluctant with their statements. Only a select group of departments participated. For instance, the department of physiotherapy and occupational therapy was not invited, a function that will be central in a new ED. Their presence might have provided additional perspectives to assess the full potential of future patient pathways and oilcloth sessions as an implementation strategy. Another limitation is that our study is the first to examine oilcloth sessions as an implementation strategy and the first to use Biggs and Tang's theoretical model for alignment. This makes it impossible to compare our results with other similar studies, which may decrease the reliability of the results. Despite this, our results highlight the relevance of securing alignment between elements in implementation strategies, an aspect that seems somewhat overlooked in the literature, which usually focuses on the effectiveness of strategies [73, 74].

We believe that the transferability of the study findings is quite high. This is due to use of ‘thick description’ [82] that provide a comprehensive and detailed account of the participants’ experiences with oilcloth sessions. These descriptions help provide a richer and fuller understanding of the research setting for the readers and thereby make it possible to transfer findings from the Danish setting to EDs in other countries. Also, given the use of theory to guide the analysis we think it is possible to transfer the idea of alignment to other implementation processes where especially educational implementation strategies are used.

## Conclusion

This study show that it is important to ensure alignment between elements in implementation strategies, i.e. conducting oilcloth sessions with high alignment, if the challenges that are experienced are to be overcome and the strategy experienced as useful in supporting the implementation of a new ED from the participants’ point of view.

### Implication for practice

If oilcloth sessions are to be used as an implementation strategy, it is recommended that the planning of these sessions focuses on alignment between clear objectives, suitable participants, and cases. If managers are to participate, it would be relevant to schedule pre-meetings to ensure alignment among the managers and to gain insight into contextual factors, including the situation they experience themselves to be in. We would also recommend considerations of whether a physical visit to new buildings or locations can be a help to participants who have not previously participated in oilcloth sessions. Communication of a clear plan about the purpose of oilcloth sessions as an implementation strategy before and after the sessions is essential for experiences of alignment. We believe the facilitator role should be discussed and selected based on facilitator skills. The greater the complexity, hierarchies and power involved in the implementation process, the more significant it is to select a trained facilitator. Bigg and Tang's didactic model is useful as an analytical framework when alignment is to be ensured in implementation strategies in general.

## Data Availability

The data set generated and analyzed during the current study is not publicly available as it contains potentially identifying or sensitive information that could compromise the privacy of the respondents according to regulations set out by the Danish Data Protection Agency but is available from the corresponding author on reasonable request.

## Abbreviations

ED: Emergency department
IF: Implementation facilitation
IS: Implementation science
QIF: Quality Implementation Framework
